# Upper gastrointestinal hemorrhage and thoracic aortic aneurysm rupture as presenting signs of Behçet disease: A case report

**DOI:** 10.1097/MD.0000000000017455

**Published:** 2019-10-11

**Authors:** Li-Fei Yu, Li-Wei Guo, Jiang-Ping He, Bin Yang, Chuang-Hua Chen, Jie Jin

**Affiliations:** a Department of Infectious Diseases, Affiliated Hangzhou First People's Hospital, Zhejiang University School of Medicine, Hangzhou; b Department of Hepatology, Qianfoshan Hospital Affiliated to Shandong University, Jinan, Shandong; c Department of Endocrinology and Rheumatology; d Department of Radiology; e Department of Ultrasound, Affiliated Hangzhou First People's Hospital, Zhejiang University School of Medicine, Hangzhou, China.

**Keywords:** Behçet disease, esophageal ulcer, nonhealing incision, thoracic aortic aneurysms rupture

## Abstract

**Introduction:**

Behçet disease (BD) is an autoimmune disorder characterized by oral aphthosis, genital aphthosis, ocular lesions, and arthritis. However, other fatal complications are often misdiagnosed, which implies that the early diagnosis of the disease is important for a good prognosis.

**Patient concerns:**

A 51-year-old man complained of hematemesis for 5 hours and syncope once.

**Diagnosis:**

BD as demonstrated by esophageal ulcer and aortic aneurysm rupture.

**Interventions:**

Surgeries were conducted to repair the thoracic aortic aneurysm, proton-pump inhibitor was used to reduce acid secretion, antibiotics were applied for anti-infective therapy, and immunosuppressor was administered to control the injuries of BD.

**Outcomes:**

The patient was discharged and his medication dosage was reduced gradually until the minimum maintenance dose. In the follow-ups, the gastric ulcer and vascular aneurysm were not found.

**Conclusion:**

We presented a rare case of BD with the concurrence of huge esophageal ulcer and thoracic aortic aneurysms rupture, which helped us to diagnose BD at the early stage, while confronting atypical manifestations.

## 1 Introduction

Behçet disease (BD) is characterized by a set of clinical manifestations, including relapsing oral aphthosis (OA), genital aphthosis, ophthalmological manifestations, skin manifestations, and multisystem involvement.^[1]^ Vascular manifestation, although not seen in all patients, is one of the most important features of BD. Nearly 7.7% Chinese BD patients have been reported to have vascular lesions, and 8.8% have been reported to have gastrointestinal (GI) manifestations.^[2]^ The symptoms of patients with GI involvement vary from retrosternal pain, hematemesis to gastritis, and diarrhea, depending on the location of ulcers. Vascular manifestations include venous and arterial manifestations, demonstrated by superficial and deep vein thrombosis, arterial thrombosis, and aneurysms; however, only 4.5% of the cases in China have been diagnosed with aneurysms.^[3,4]^ Normally, immunosuppressive medication is administered to control BD symptoms, but in case rare and deadly complications, such as aneurysm rupture, occur, immediate surgery is recommended.^[5–8]^ Unfortunately, the best opportunity for surgery is often missed because of the delayed diagnosis of BD and lack of awareness of this fatal complication. Up to now, there were no data showing the frequency of the concurrence of vascular and GI involvement, and no published paper reporting such cases. Here we report a rare case of BD presented by the concurrence of upper GI hemorrhage and thoracic aortic aneurysm rupture, which was treated effectively.

## 2 Case presentation

**Figure 1:**
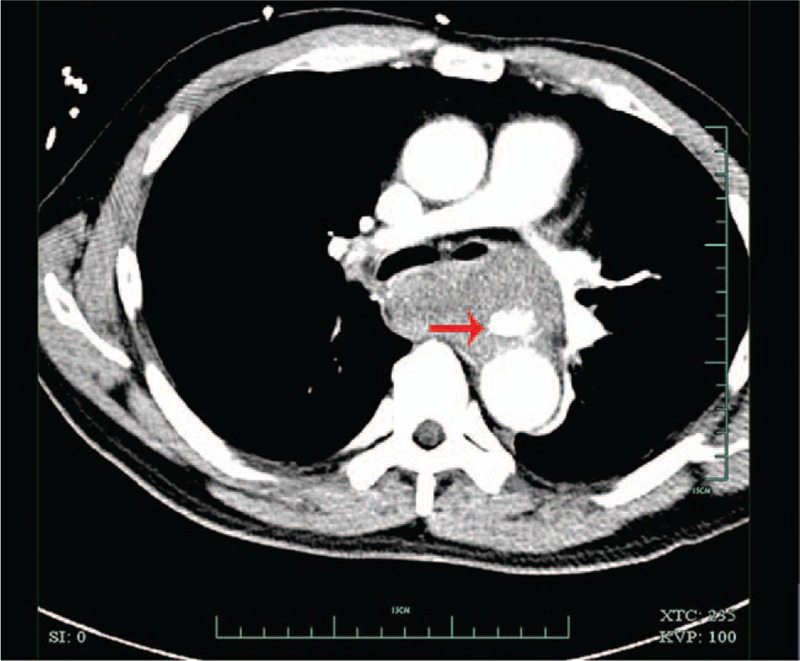
Thoracic aorta computed tomography scan before surgery. Remarkable aneurysm was detected in the thoracic aorta, which ruptured into the mediastinum (red arrow).

**Figure 2:**
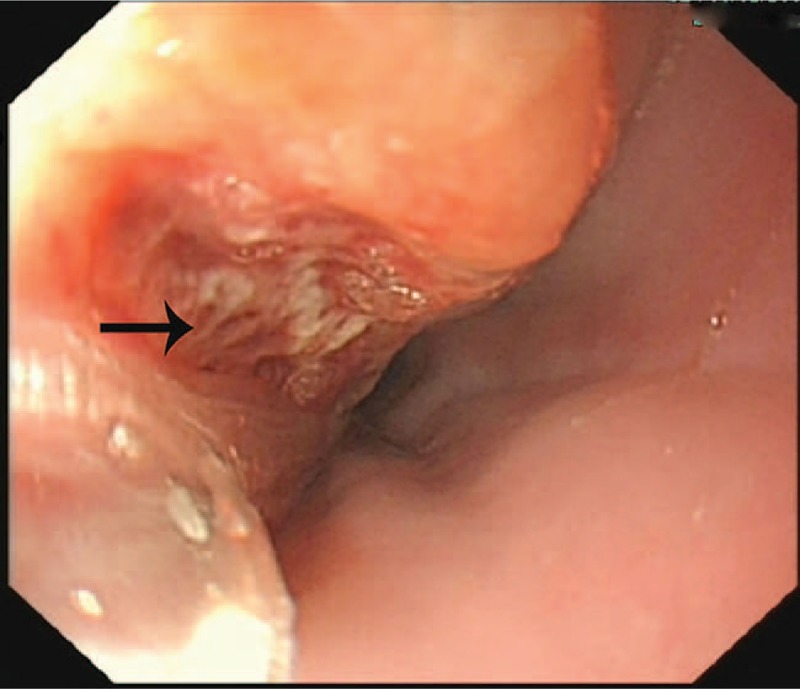
Gastroscope of the patient. A huge penetrating ulcer was found in the esophagus (black arrow).

A 51-year-old man complaining of hematemesis for 5 hours was admitted to the hospital. The patient also complained of syncope once outside the hospital. He denied having epigastric pain or nausea, and had no history of hypertension or chronic liver disease. He reported no chest pain, shortness of breath, or cough. He denied history of any hereditary disease. Physical examination confirmed the following: normal body temperature (37.0°C), blood pressure of 123/70 mm Hg, heart rate of 80 beats per minute and respiratory rate of 20 breaths per minute. The laboratory test results such as leukocytes’ count and hemoglobin were within normal range. The patient had a thoracic aorta computed tomography (CT) scan, which surprisingly demonstrated “thoracic aortic aneurysms rupture” (Fig. 1). Presurgical examinations were performed immediately and after preclusion of contraindications, the patient underwent a surgery involving the right femoral artery puncture, aortic angiography, and endovascular repair for thoracic aortic aneurysm. Subsequently, gastroscopy was also performed, which revealed a huge esophageal-penetrating ulcer (Fig. 2). The patient was treated with proton-pump inhibitor (PPI) and other supporting medications for nearly 4 weeks and was discharged from the hospital.

**Figure 3:**
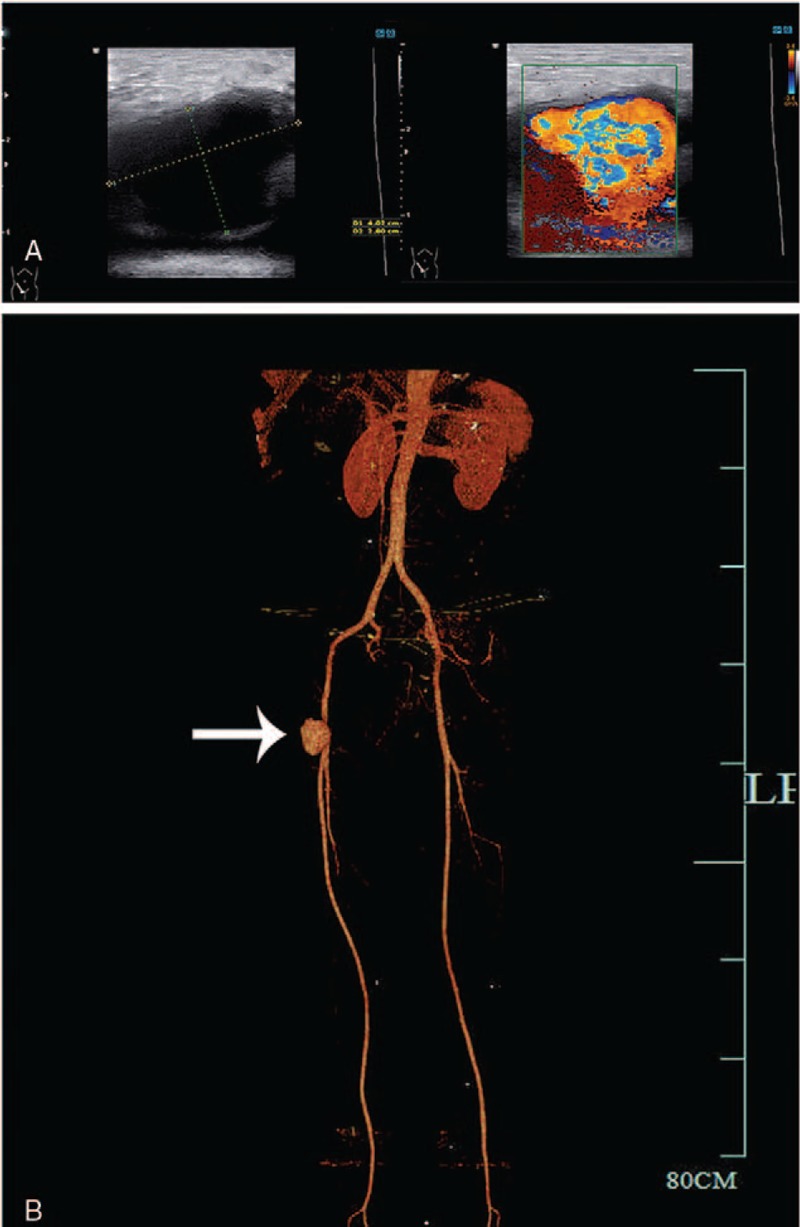
Ultrasonography and CTA in lower extremity arteries. (A) A pseudoaneurysm approximately 4 × 3 cm was confirmed by ultrasonography. (B) A remarkable pseudoaneurysm was also suggested by CTA in lower extremity arteries (arrow). CTA = computed tomography angiography.

**Figure 4:**
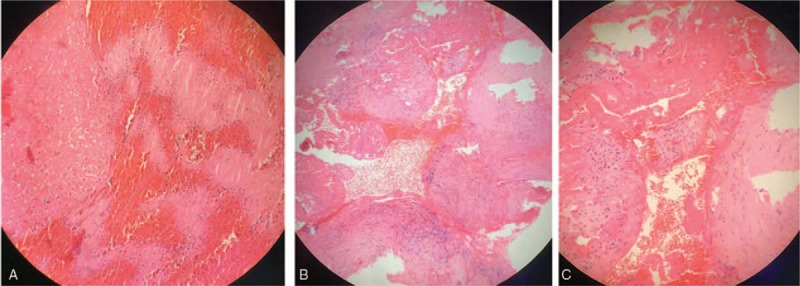
Tissue pathology of the pseudoaneurysm. (A) Hematoxylin and eosin staining of the cavity revealed thrombus with infiltration of inflammatory cells. (B and C) Hematoxylin and eosin staining of the aneurysm wall. No vascular endothelial cells were observed.

**Figure 5:**
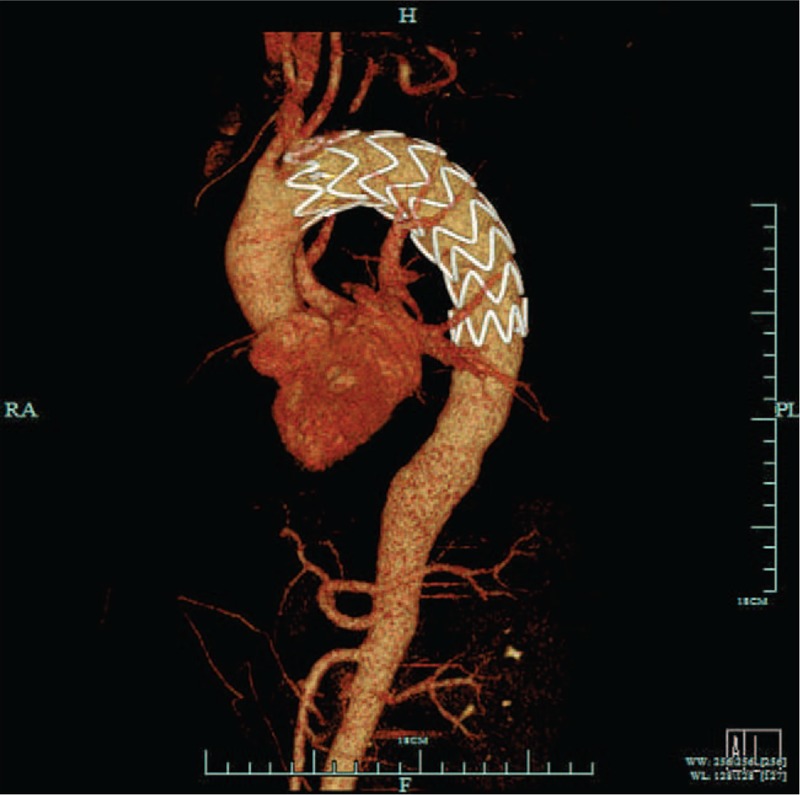
Thoracic aorta CTA of the patient after surgery. The aorta stent is displayed, which was found in the correct place. CTA = computed tomography angiography.

One month later, however, the patient was readmitted to the hospital after complaining of a painful mass in the puncture point in the right femoral artery and recurrent fever (T_max_ was 38.6°C) at the same time. Ultrasonography and CT angiography (CTA) in lower extremity arteries suggested pseudoaneurysm (Fig. 3), which was confirmed by tissue pathology (Fig. 4). The main laboratory test indices were as follows: leukocytes 5.0 × 10^9^/L, neutrophil granulocytes 79.6%, hemoglobin 109 g/L, platelets 325 × 10^9^/L, hypersensitive C-reactive protein (CRP) 105 mg/L. Reexamination of thoracic aorta by CTA revealed postoperative changes in the thoracic aortic aneurysm cavity (Fig. 5). Echocardiography showed no neoplasm in the valves. Postoperative infection was the first impression; therefore, piperacillin-tazobactam was administered at the dosage of 4.5 g once every 8 hours to control the infection. The second surgery was performed to resect the pseudoaneurysm and repair the right femoral artery. Unfortunately, the incision did not heal as expected. A large area of redness appeared around the wound and the patient reported intense pain. Upon the removal of suture, about 15 mL of dark brown liquid drained through the incision, which was suggestive of incision infection. Therefore, we applied vancomycin at the dosage of 1 g once every 12 hours, and changed the dressing every day. Nevertheless, low-grade fever persisted (37.7°C–38.0°C) for over a month, while 2 sets of blood cultures and excretion cultures had no positive findings. Therefore, the patient underwent the third surgery named “right inguinal debridement and vacuum sealing drainage negative pressure closed drainage and femoral thin muscle flap transfer repair.” However, the temperature did not drop to normal, and the incision still did not heal. Routine blood test showed white blood cell count 4.7 × 10^9^/L, neutrophil granulocyte 60.9%, hemoglobin 104 g/L, platelet 45 × 109/L, CRP 48 mg/L, and erythrocyte sedimentation rate (ESR) 55 mm/hour.

**Figure 6:**
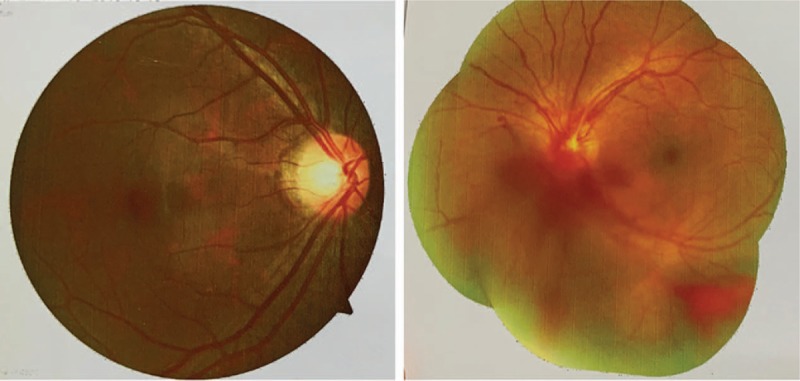
Fundus examinations of the patient showed uveitis. Retinal angiopathy and vitreous hemorrhage can be observed in the fundus image.

After careful reexamination of the medical history of the patient, we noticed that the patient also complained of relapsed OA (more than 3 episodes in a year) and blurred vision. Fundus examination was arranged, and the result showed uveitis (Fig. 6). To sum up, the patient suffered from thoracic aortic aneurysm, esophageal ulcers, persistent fever, and vascular pseudoaneurysms formation; and he was finally diagnosed with BD according to the International Criteria for BD (ICBD, Table 1).^[9,10]^ Subsequently, 40 mg of methylprednisolone and 100 mg of azathioprine (50 mg twice per day) was applied to the patient on daily basis. Soon, the temperature returned to normal and the right inguinal incision healed. He was discharged, and his medication dosage was reduced gradually under the supervision of our physician until the maintenance dose of 4 mg/day (methylprednisolone) and 50 mg/day (azathioprine). Now, his condition is stable and has not had a relapse ever since, and laboratory results such as ESR and CRP are normal in the follow-up tests.

**Table 1:**
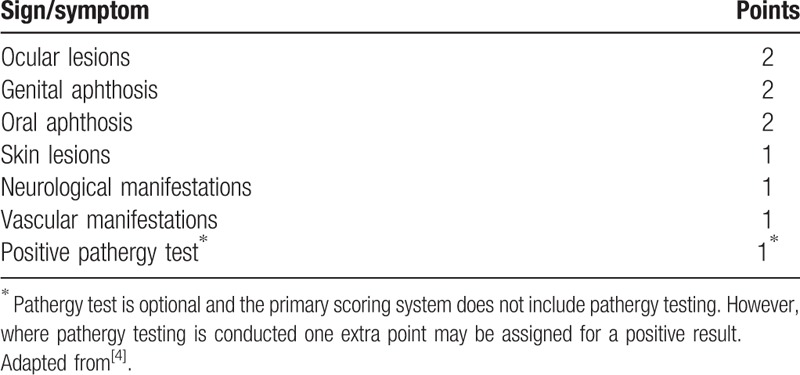
International Criteria for Behçet disease – point score system: scoring ≥4 indicates Behçet diagnosis.

## 3 Discussion

The patient got 5 points according to ICBD, so the disease diagnosis was established. During the diagnoses and treatment, the most difficult part was to distinguish whether the aneurysm and gastric ulcer were manifestation of BD or isolated events because the treatment would have been different. According to previous reports, BD could affect various types of vessels to cause different kinds of lesions, but upper GI hemorrhage and thoracic aortic aneurysm rupture as presenting signs of BD is rare, let alone the concurrence of the two.^[11–13]^ The common sites of aneurysm in BD have been reported as abdominal aortic aneurysm, carotid artery aneurysm, and lower extremity aneurysm^[11]^; however, thoracic aortic aneurysm has been barely reported.

The patient in our case had no history of hypertension, dyslipidemia, or other related high-risk comorbidities. However, he suffered from relapsed fever and infection during the course of disease and pseudoaneurysm formation after interventional surgery, suggesting that his immune system was disturbed and vascular structure and function was impaired. Vascular stent was used immediately to repair the aneurysm, but he did not heal well until the administration of glucocorticoid, thus, suggesting that the vascular lesion was not an isolated event but was related to BD. However, owing to the involvement of GI tract and vascular lesion in BD, the treatment was more complicated. The symptoms tended to relapse, so close follow-ups were needed to monitor the course of disease. Usually, mediate to large dosage of glucocorticoid as well as immunosuppressive agents should be administered, and laboratory tests such as CRP, ESR, hemoglobin, and other auxiliary examinations like endoscopy and CT should be reviewed.^[14]^ The medication dosage should be reduced gradually and according to patients’ follow-up results, relevant medical measures such as PPI or surgery should be conducted, if peptic ulcer or aneurysm is discovered. In the follow-up tests of our patient, the laboratory results were within normal range, so we gradually reduced his mediation to the minimum maintenance dose, and excitingly, no aneurysm was detected ever since.

To conclude, if a patient is discovered to have pseudoaneurysm and esophageal ulcer at the same time, along with nonhealing wounds and persistent fever after antibiotic treatment, the possibility of BD should be considered. Besides, detailed medical history and careful physical examination is of great value in occult onset patients. The patient in our case responded well to the immunosuppressive therapy. During the follow-up treatment, we should focus more on the side effects of glucocorticoid and azathioprine as well as the complications of the surgeries, and carefully adjust medication according to his/her condition.

## Acknowledgment

We would like to thank Editage (www.editage.cn) for English language editing.

## Author contributions

**Data curation:** Li-Fei Yu, Jiang-Ping He, Li-Wei Guo, Bin Yang, Chuang-Hua Chen.

**Investigation:** Jie Jin.

**Supervision:** Jie Jin.

**Writing – original draft:** Li-Fei Yu.

**Writing – review & editing:** Jiang-Ping He.
